# The role of Iranian medicinal plants in experimental surgical skin wound healing: An integrative review

**DOI:** 10.22038/ijbms.2019.32963.7873

**Published:** 2019-06

**Authors:** Amin Derakhshanfar, Javad Moayedi, Ghazal Derakhshanfar, Ali Poostforoosh Fard

**Affiliations:** 1 Diagnostic Laboratory Sciences and Technology Research Center, School of Paramedical Sciences, Shiraz University of Medical Sciences, Shiraz, Iran; 2 Center of Comparative and Experimental Medicine, Shiraz University of Medical Sciences, Shiraz, Iran; 3 Vice Chancellery for Research Affairs, Shiraz University of Medical Sciences, Shiraz, Iran

**Keywords:** Experimental, Iranian medicinal plants, Skin, Surgical, Wound healing

## Abstract

**Objective(s):**

Wounds are physical injuries that cause a disturbance in the normal skin anatomy and function. Also, it has a severe impact on the cost of health care. Wound healing in human and mammalian species is similar and contains a complex and dynamic process consisting of four phases for restoring skin cellular structures and tissue layers. Today, therapeutic approaches using herbal medicine have been considered. Although the benefits of herbal medicine are vast, some medicinal plants have been shown to have wound healing effects in different experimental studies. Therefore, the current review highlights information about the potency of herbal medicine in the experimental surgical skin wound healing.

**Materials and Methods:**

Electronic database such as PubMed, Google Scholar, Scopus, and Medscape were searched for Iranian medicinal plants with healing activity in experimental surgical skin wounds. In this area, some of the most important papers were included.

**Results:**

There are numerous Iranian medicinal plants with skin wound healing activity, but clinical application and manufacturing are very low in comparison to the research volume.

**Conclusion:**

In normal instances, the human/animal body usually can repair tissue damage precisely and completely; therefore, the utilization of herbs is limited to special conditions or in order to accelerate the healing process.

## Introduction

The skin is the largest organ of the body and is the first line defense against injury and plays critical roles in maintaining homeostasis (1). A wound is a skin injury that is made by physical, chemical, or microbiological infections at all ages (2). The methods of managing wounds have changed dramatically in recent decades and wound healing is now a challenging global clinical problem. The primary goal is to heal the wound as fast as possible (3). The process of this natural restorative response to tissue injury is very complex, and four precisely and highly programmed phases are involved. They comprise hemostasis, inflammation, proliferation, and tissue remodeling that must occur in the proper sequence and time frame to create optimal wound healing (1, 3). Some factors such as oxygenation, infection, age, sex, hormones, stress, diabetes, obesity, medications, alcoholism, smoking, and nutrition can affect the wound healing process, causing improper or impaired tissue repair (1, 4).

Animal models are essential biological tools for ensuring the safety and efficacy of human drugs. Humans and animals have similar skin characteristics, therefore, an accurate model of human wound healing was simulated in animals (5). All animal models have been widely used in wound healing studies. Various animal wound models, especially rats and mice, are used to reflect human wound healing problems. Despite species differences, availability and relatively low cost allow rodents to serve as a valuable research tool (6, 7).

Herbal products including plant-derived extracts have long been used in the treatment of wounds and found to possess significant effects on wound healing (3). In recent years, the use of natural products especially those derived from plants has increased. World health organization estimated that more than 80% of the world’s population relies on traditional medicines for various skin diseases (8). On the other hand, there are many reports about medicinal plants affecting the wound healing process. Regardless of production method, formulation, component, antibacterial activity, and possible side effects, the efficacy of herbal extracts has been evaluated in excision, incision, dead space, and burn wound models (3). It has been estimated that 1–3 % of the modern drugs in use are for the treatment of wounds and skin disorders compared to one-third of all traditional medicines (8). 

In Iranian herbal remedies, plant extracts have been used for treating a wide range of illnesses. Iran is one of the primary loci for medicinal plants, where now over 8000 species of flowering plants are growing (9). However, despite abundance of papers by Iranian investigators in the field of experimental surgical skin wound healing and herbal remedies, only a few are practically applied in the treatment of patients (unpublished data). Therefore, this review focuses on plants with medicinal properties and represents the significant efficacy of Iranian herbal products in experimental and surgical procedures and discusses their utilization in clinical medicine.

## Materials and Methods

We have conducted extensive research in PubMed, Google Scholar, Scopus, and Medscape covering years 2000 to 2017 using keywords including experimental, surgical, skin wound healing, Iranian herbal medicine, Iranian ethnopharmacology, and Iranian medicinal plants. Then, the most important papers in the field of experimental surgical procedure were selected. This study was performed based on Iranian articles and Iranian medicinal plants; therefore, any non-Iranian articles were excluded.

## Results

***Peganum harmala***

The extract of *Peganum harmala* which is commonly called wild rue was studied on skin defects and found to be effective in wound healing, causing a significant decrease in the epithelial gap and an increase in tensile strength of the wounds (10).

***Quercus brantii***

Evaluation of the efficacy of *Quercus brantii* or balut on the full-thickness wounds shows significant healing improvement in wound contraction, epithelialization period, hydroxyproline content, and tensile strength as compared with the control group (11).

***Quercus persica***

*Quercus persica* also called balut is found in Zagrossian region of Iran. The efficacy of methanolic extract of *Q. persica* as an antibacterial agent was confirmed in all concentrations, but it is significant in concentrations of 50 and 75 mg/ml. Besides, this extract had a wound healing potency and significant impact on epithelialization and reduction of the epithelial gap (12).

***Elaeagnus angustifolia***

The wound healing efficacy of* Elaeagnus angustifolia *or oleaster was tested on full-thickness skin wounds and showed a significant contraction and hydroxyproline increase after 15 days, which means it is probably useful in wound healing (1). In addition, the oleaster leaves water-soluble extract shows a pro-healing effect on experimental wounds and seems to be an available herbal preparation with a reasonable cost (13).

**Table 1:** Some Iranian medicinal plants with healing activity on experimental surgical skin wounds

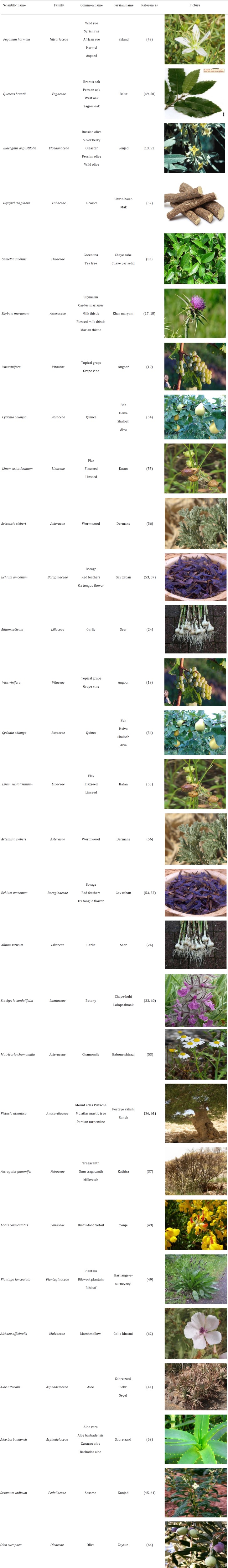

**Table 2:** The animal species discussed in this review

**Animal model**	**Number of studies**	**References**
**Mice**	2	(32, 46)
**Rats**	27	(1, 2, 8, 10-14, 16-18, 22, 23, 30, 31, 33, 34, 36-39, 41-45, 47)
**Rabbits**	9	(5, 19-21, 25, 26, 28, 40)
**Dogs**	1	(24)
**Calves**	1	(15)

**Table 3:** Kinds of wounds discussed in this review

**Kind of wound**	**Number of studies**	**References**
**Excision**	29	(1, 5, 8, 10-15, 17-23, 25, 26, 28, 30, 31, 33, 36-40, 42, 43)
**Incision**	11	(2, 16, 24, 32, 34, 36, 41, 44-47)

***Glycyrrhiza glabra***

The effect of *Glycyrrhiza glabra* root extract (licorice) was tested on skin wounds of rats and rabbits. The results suggested that both licorice root extract and the hydro-alcoholic extract were able to cause the contraction of the wound, epithelialization, and infiltration of fibroblasts (5, 14). Moreover, a combination of licorice with sesame oil was an effective remedy for wound healing in female Holstein calves (15).

***Camellia sinensis***

The wound healing activity of* Camellia sinensis* also referred to as green tea was evaluated in Wistar rats; the ethanolic extract could accelerate the healing process (16).

***Silybum marianu*** **m**

*Silybum marianum,* which is commonly called silymarin was supposed to have anti-inflammatory and antioxidant activity (17). The application of silymarin on full-thickness wounds in Wistar rats causes increased in the fibroblast count, collagen synthesis, and wound contraction (17). Also, it improved the morphological, biochemical, and biomechanical properties of full-thickness wounds in rats (18).

***Vitis vinifera***

Wound healing activity of the hydro-alcoholic extract of *Vitis vinifera* (topical grape) seed was assessed on 20×20 mm square-shaped excision wounds in rabbits. The results show that topical grape was able to increase hydroxyproline and tensile strength of the wound and improve the healing process (19).

***Cydonia oblonga***

The aqueous extracts of *Cydonia oblonga *known as quince were prepared and applied on experimental wounds in rabbits, which was able to cause a significant acceleration in wound healing (20). Moreover, quince seed mucilage in 10–20% concentration has a good potential for promoting wound healing in rabbit (21).

***Linum usitatissimum***

The effect of *Linum usitatissimum *oil*,* which is known as flaxseed oil was evaluated on experimental incisions in rats and the results showed suppression of inflammatory process and wound healing acceleration (2).

***Artemisia sieberi***

Wormwood or* Artemisia sieberi,* which is mainly found in the Yazd province of Iran was extracted and applied on punch skin wounds. Results suggested that it could increase the fibroblastic population in the wound site and also cause wound contraction and angiogenesis (22).

***Echium amoenum***

The borage or *Echium amoenum* is another plant that has a significant level of gamma-linolenic acid, alpha-linolenic acid, and delta-6 fatty acid. The application of this plant on punch wounds in rats shows a decrease in wound size, and it could accelerate wound healing (23).

***Allium sativum***

Aqueous extract of garlic (*Allium sativum*) was applied to rectangular wounds in dogs, which resulted in a decrease in the epithelial gap and collagen synthesis (24).

***Punica granatum***

Ethanolic extract of *Punica granatum*, also called pomegranate, was prepared and applied on incisions in Wistar rats, which significantly reduced the wound size (8).

***Achillea kellalensis***

The aqueous extract of *Achillea kellalensis* (yarrow) showed significant wound healing activity on experimental full-thickness excision wound in male Wistar rats (8).

***Achillea millefolium***

The wound healing effect of the hydroalcoholic extract of *Achillea millefolium* was evaluated on full-thickness excision wounds in rabbits, and the concentration of 5% could accelerate the collagenation and proliferation of wound healing (25).

***Verbascum thapsus***

*Verbascum* *thapsus *is a traditional remedy for wound healing, which is commonly called mullein. The application of this plant extract at the concentration of 20% shows a significant wound healing activity in rabbits, making it a promising drug for the future (26).

***Hypericum perforatum***

Saint John’s wort (*Hypericum* *perforatum*) can improve tissue regeneration by enhancing fibroblast proliferation (27). *H. perforatum *hydro-alcoholic extract was prepared and applied to rabbit wounds. As a result, the healing time was decreased, which means this plant could have a potential for wound healing acceleration (28). Moreover, *H. perforatum* can speed up cesarean wound healing with minimal scar formation (29).

***Myrtus communis***

The leaves of this plant known as myrtle could cause enhancement of wound contraction and fibroblast cell proliferation, which lead to wound healing (30).

***Malva sylvestris***

In rural areas of Iran,* Malva* *sylvestris* (common mallow) is used for the treatment of burn and cut wound healing. Experimental findings in rats demonstrate that extract of *M. sylvestris* effectively stimulates wound contraction (31). The beneficial effects of aqueous extract of *M. sylvestris* on the wound healing process were also evaluated in BALB/c mice (32).

***Stachys lavandulifolia***

The wound healing potency of *Stachys* *lavandulifolia *locally known as betony was investigated in rats using the excision wound model. The extract-treated animals showed about 95% reduction in the wound area compared with 92% for nitrofurazone as a standard drug (33).

***Matricaria chamomilla***

The extract of *Matricaria* *chamomilla* revealed an ability for wound healing in linear incisional wound model (34). The aqueous extract of *M. chamomilla *was also effective in treatment against acetic acid-induced colitis in rats (35).

***Pistacia atlantica***

*Pistacia* *atlantica*, locally known as mount atlas pistache, is used in traditional Iranian therapies for inflammatory wounds. The role of the hydro-ethanolic extract of *P. atlantica *was evaluated in excision and incision wounds in rats. The results show that *P. atlantica *ointment could shorten the inflammation phase by provoking the proliferation of fibroblasts. Moreover, it also promotes neovascularization and angiogenesis (36).

***Astragalus gummifer***

The wound healing activity of tragacanth (*Astragalus gummifer*) has been evaluated in full-thickness wounds of albino rats, and the results show skin wound contraction and healing acceleration in this model (37).

***Lotus corniculatus (L. corniculatus)***

A full-thickness rectangular wound was made in male rats to evaluate the healing effect of the hydro-ethanolic extract of *Lotus corniculatus*. The results showed that in addition to anti-inflammatory and anti-microbial effects, *L. corniculatus *is more effective in the healing of the wounds (38).

***Plantago lanceolata***

The efficacy of plantain (*Plantago* *lanceolata*) in the acceleration of wound healing was also evaluated in rats. The wounds with 0.75% *P. lanceolata* extract dressing had significantly accelerated wound healing enclosure (100% healing) during 14 days (39).

***Althaea officinalis***

The effect of flower mucilage of* Althaea* *officinalis*, commonly known as marshmallow, was evaluated on experimental full-thickness wounds and the results indicated reduction in the duration of wound healing (40).

***Aloe littoralis***

The significant healing effect of *Aloe* *littoralis* raw mucilaginous gel was shown in Wistar rats (41).

***Aloe barbandensis***

Both aqueous extract and gel of *Aloe* *barbandensis*, known as Aloe vera, were also evaluated in experimental wounds in rats, which showed wound contraction and healing acceleration (42, 43). Moreover, re-epithelialization and angiogenesis were significantly improved in the Aloe vera gel group with surgical incisions (44).

***Sesamum indicum***

Sesame oil extract, which contains sesamin and sesamolin was tested on wounds in Wistar rats, which led to significant wound contraction and a decrease in wound length and healing time (45).

***Olea europaea***

Oleuropein is the main component of the olive leaf extract (*Olea* *europaea*). The application of oleuropein to incision wounds in BALB/c mice showed a significant increase in the contraction of wounds (46).

**Herbal marine compound**

Herbal marine compound is a drug of marine herbal origin, and various concentrations of this remedy were used for investigation of wound healing in rats. The results suggest that the herbal marine compound could have a dose-dependent effect on healing and contraction of the wound (47). Table 1 shows Iranian medicinal plants with healing activity on experimental surgical skin wounds. Tables 2 and 3 indicate animal species and kind of wounds in the present review, respectively.

## Discussion

Wound is a disruption of the anatomical and functional integrity of the living tissue (48). Immediately, after a wound develops, the process of healing begins. Injured tissue goes through four temporal phases to repair the wound: hemostasis, acute inflammation, proliferation (granulation), and remodeling (maturation, contraction). The success of skin wound healing is often determined by whether the process occurs via first or second intention healing. First intention healing in the skin occurs when the edges of a wound site are directly apposed and re-attached and heal to each other rapidly. The wound lacking such close, intimate apposition is termed second intention healing (4, 49, 50). It seems some authors did not pay attention to differences between incision and excision wounds (2, 26). Incision means surgical cut into body tissues, while an excision involves taking the tissue out. So, the application of the term “incision” when the tissue is removed is not correct.

Plants have always been one of the most available resources for treating diseases. Persians were pioneers in applying plants for medicinal treatment. Iran has 11 climates out of the 13 world climates and has 7500–8000 plant species (51). The healing properties of these plant remedies are achieved through their secondary metabolites such as alkaloids, glycosides, flavonoids, saponins, tannins, carbohydrate, and essential oils via anti-inflammatory, anti-oxidant, and anti-bacterial activity, angiogenesis, growth factor enhancement, cellular proliferation, collagen synthesis, re-epithelialization, and tissue remodeling (48). However, there is much still unknown about the role of plants in the treatment of wounds. In this regard, the major limitation is the paucity of clinical studies and the complexity of chemical constituents. As a rule, medicinal plants must be analyzed to identify the main active components (52).

## Conclusion

It should be noted that in normal instances the human/animal body usually can repair tissue damages wholly and precisely. Therefore, the utilization of herbs is limited to particular conditions or in order to accelerate the healing process. Now, two fundamental questions must be answered:

1) To what extent the results of these studies can be applied to humans in clinical treatments?

2) To what extent the results of research will lead to the manufacturing of a new drug?

To answer the questions mentioned above will hopefully trigger further research in Iran.
